# Growth Hormone-Releasing Hormone Antagonist JV-1-36 Suppresses Reactive Oxygen Species Generation in A549 Lung Cancer Cells

**DOI:** 10.3390/endocrines3040067

**Published:** 2022-12-09

**Authors:** Khadeja-Tul Kubra, Mohammad S. Akhter, Kaitlyn Apperley, Nektarios Barabutis

**Affiliations:** School of Basic Pharmaceutical and Toxicological Sciences, College of Pharmacy, University of Louisiana Monroe, 1800 Bienville Drive, Monroe, LA 71201, USA

**Keywords:** inflammation, lung injury, endothelium, oxidative stress

## Abstract

Growth hormone-releasing hormone (GHRH) and its receptors are expressed in a variety of human cancers, and have been involved in malignancies. GHRH antagonists (GHRHAnt) were developed to suppress tumor progression and metastasis. Previous studies demonstrate the involvement of reactive oxygen species (ROS) in cancer progression. Herein, we investigate the effect of a commercially available GHRH antagonist, namely JV-1–36, in the redox status of the A549 human cancer cell line. Our results suggest that this peptide significantly reduces ROS production in those cells in a time-dependent manner and counteracts H_2_O_2_-induced ROS. Our study supports the anti-oxidative effects of JV-1–36 and contributes in our knowledge towards the in vitro effects of GHRHAnt in cancers.

## 1. Introduction

Growth hormone-releasing hormone (GHRH) is a hypothalamic hormone which regulates the release of growth hormone (GH) from the anterior pituitary gland. It consists of 44 amino acids and has been involved in mitogenic processes of a diverse variety of human malignancies including breast, lung, ovary, and prostate cancers [1]. It can directly bind to the pituitary-type GHRH receptor (GHRH-R), a member of the class B G-protein-coupled receptor (GPCR) family [2]. That binding activates signaling cascades which promote cancer aggression and inflammation, at least in part due to insulin-like growth factor 1 (IGF1) release [1].

GHRH-R shares homology with the vasoactive intestinal peptide (VIP), pituitary adenyl cyclase activating polypeptide, and calcitonin receptors. The NH_2_-terminal 29 amino-acid sequence of GHRH preserves its biological activity [3]. GHRH-R contains seven hydrophobic transmembrane domains and regulates cell cycle progression and differentiation [4]. The proliferation rate of breast, prostate, and lung cancer cells is suppressed when intrinsic GHRH production is silenced due to small interfering RNA specifically designed for this neuropeptide [5]. Moreover, active receptors for GHRH were detected in surgical specimens of human prostate, ovarian, endometrial, adrenal, and pancreatic cancers [6]. Considering that GHRH is ectopically expressed, it was suggested that it can act as an autocrine/paracrine growth factor in tumor development [7].

Splice variants (SVs) of GHRH-R were detected in a diverse variety of human and animal tissues, including the lungs [8]. Although several SVs were characterized, SV1 is the most abundantly expressed and possesses ligand-dependent and -independent activities [9]. Pancreatic, colon, and gastric cancer cells expressed mRNA for both GHRH and SV1 [10]. That discovery shed light onto the mechanisms mediating the anti-cancer activities of GHRHAnt [1].

Reactive oxygen species (ROS), a byproduct of cellular respiration, can deteriorate DNA, proteins, and lipids [11]. Those oxidants potentiate angiogenesis, metastasis, and survival. ROS can trigger mitogen-activated protein kinase/extracellular signal-regulated protein kinases 1/2 (MAPK/ERK1/2), p38, c-Jun N-terminal kinase (JNK), phosphoinositide-3-kinase/ protein kinase B (PI3K/Akt), nuclear factor-kappa B (NF-κB), matrix metalloproteinases (MMPs), and vascular endothelial growth factor (VEGF) [12]. GHRHAnt inhibit those cascades, which in turn suppress ROS generation [13,14]. Our study investigates for the first time the anti-oxidative effects of JV-1–36 in the H_2_O_2_-induced A549 ROS generation. The outcomes substantiate ongoing research on the beneficial effects of GHRHAnt in lung maintenance and function, and provide new information in the corresponding context.

## 2. Materials and Methods

### 2.1. Reagents

Anti-mouse IgG horseradish peroxidase (HRP)-linked whole antibody from sheep (95017–554), anti-rabbit IgG HRP-linked whole antibody from donkey (95017–556), nitrocellulose membranes (10063–173), radioimmunoprecipitation assay (RIPA) buffer (AAJ63306-AP), 2′,7′-dichlorofluorescein diacetate (DCFDA) (10180–506), and 3-(4,5-Dimethylthiazol-2-yl)-2,5-diphenyltetrazolium bromide (MTT) (BT142015–5G) were obtained from VWR (Radnor, PA, USA). The GHRH-R antibody (ab28692) was purchased from Abcam (Cambridge, MA, USA). GHRHAnt JV-1–36 (031–23) was obtained from Phoenix pharmaceuticals Inc. (Burlingame, CA, USA), and H_2_O_2_ (H1009) from Sigma-Aldrich (St Louis, MO, USA).

### 2.2. Western Blot Analysis

Cell proteins were isolated with RIPA buffer. Equal protein amounts were separated by sodium dodecyl sulfate (SDS–PAGE) Tris-HCl gel electrophoresis. Wet transfer was used to pass the proteins onto nitrocellulose membranes, which were incubated for 60 min at room temperature in 5% nonfat dry milk, so to cover non-specific binding sites. Those membranes were then exposed overnight (4 °C) to appropriate primary antibodies (1:1000). The signal for the immunoreactive proteins was developed by using the corresponding secondary antibodies (1:2000). Chemiluminescent substrate (VWR, Radnor, PA, USA) was used to develop the signal, visualized in a ChemiDoc Touch Imaging System from Bio-Rad (Hercules, CA, USA). The β-actin antibody was used as a loading control.

### 2.3. Cell Culture

Human lung cancer cells A549 and cervical cancer cells HeLa were obtained from ATCC (Manassas, VA, USA). They were cultured in DMEM (VWRL0101–0500) medium supplemented with 10% fetal bovine serum and 1X penicillin/streptomycin. Cultures were maintained at 37 °C in a humidified atmosphere of 5% CO_2_–95% air. All reagents were purchased from VWR (Radnor, PA, USA).

### 2.4. Measurement of Cell Viability

A549 and HeLa cells were seeded onto 96-well culture plates (10,000 cells/well) in complete growth media, and were treated with GHRHAnt (0.01–15 μM) and H_2_O_2_ (0.001–2 mM) for 8 hours (h). After that time-point, the media was replaced with serum-free media containing 5 mg/mL MTT, and incubated for 3.5 h. DMSO (100 μL/well) was added to dissolve MTT crystals, and 15 min later, absorbance was measured (570 nm) utilizing the SPECTROstar Nano^®^ Absorbance Plate Reader by BMG LABTECH.

### 2.5. Cell Treatment and ROS Measurement

Cells were seeded onto a 96-well plate at a density of 1 × 10^4^ cells/well, and were exposed to vehicle (0.1% DMSO) or GHRHAnt (1 μM) for 4, 8, 16, or 24 h. In another set of experiments, cells were treated with vehicle (0.1% DMSO) or GHRHAnt (1 μM) for 8 h, before their exposure to vehicle (PBS) or H_2_O_2_ (0.1 mM) for 8 h. After those treatments, the cells were incubated with 25 μM of DCFDA for 45 min. The fluorescence intensity was measured by the fluorescence plate reader (Synergy H1 Hybrid Multi-Mode Reader from Biotek) using the excitation wavelength of 485 nm and emission wavelength of 535 nm.

## 3. Results

### 3.1. Expression of GHRH-R in Cancer Cells

**Figure 1.**
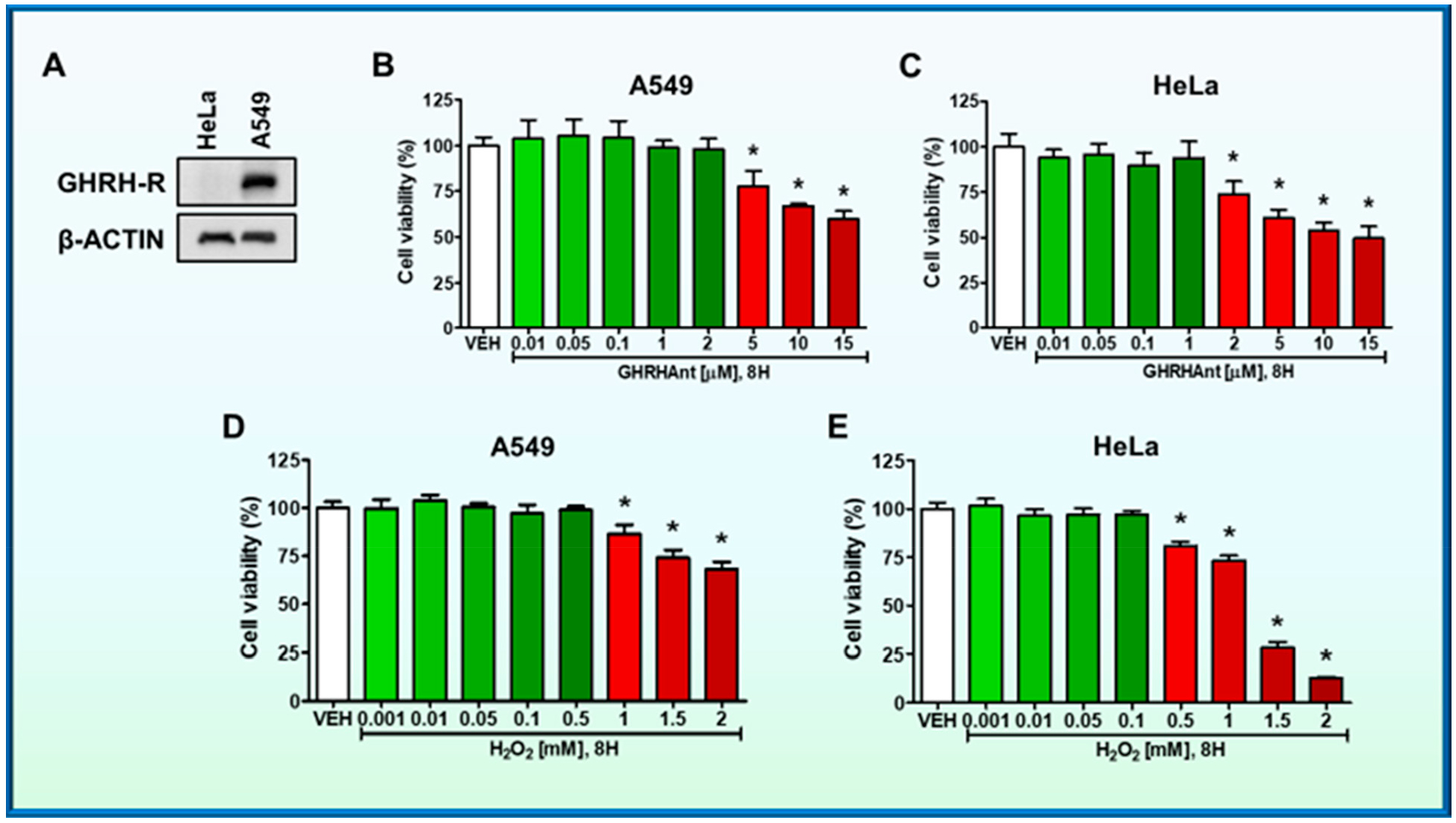
(**A**) Western blot analysis of GHRH-R and β-actin in A549 and HeLa cells. The β-actin was used as a loading control. (**B**) A549 cells were seeded onto a 96-well plate (10,000 cells on each well) and were treated with vehicle (VEH) (0.1% DMSO) or GHRHAnt (0.01–15 μM) (8 h); and (**D**) VEH (PBS) or H_2_O_2_ (0.001–2 mM) (8 h). MTT assay was used to assess cell viability. n = 3 per group. Means ± SEM. (**C**) HeLa cells were seeded onto a 96-well plate (10,000 cells/well) and were incubated with VEH (0.1% DMSO) or GHRHAnt (0.01–15 μM); and (**E**) VEH (PBS) or H_2_O_2_ (0.001–2 mM) (8 h). GHRHAnt at 0.01–1 μM, and H_2_O_2_ at 0.001–0.1 mM did not affect HeLa viability. However, viability was reduced in cells exposed to higher concentrations of GHRHAnt (2–15 μM) or H_2_O_2_ (0.5–2 mM). The graphs represent three independent experiments. * *p* < 0.05 vs. VEH. Means ± SEM.

The expression of GHRH-R in A549 human lung and HeLa cervical cancer cells was analyzed by Western blot analysis. The HeLa cell line was used as a negative control, due to the absence of GHRH-R [15]. Our results suggest that A549 express GHRH-R (Figure 1A).

### 3.2. Effects of GHRHAnt on Cell Viability in A549 Cells

The lung cancer cells were seeded onto 96-well culture plates (10,000 cells/well) in complete growth media and were treated with either vehicle (0.1% DMSO) or JV-1–36 (0.01–15 μM). After 8 h, the media was replaced with fresh media containing 5 mg/mL MTT. After 3.5 h of incubation, DMSO was added to dissolve the MTT crystals, and absorbance was measured. Our results suggest that moderate concentrations of GHRHAnt (0.01–2 μM) do not exhibit toxicity in A549 cells. However, JV-1–36 (5–15 μM) reduced cell viability at higher concentrations (Figure 1B).

### 3.3. Effects of GHRHAnt on Cell Viability in HeLa Cells

Cells were seeded onto a 96-well plate (10,000 cells in each well) and were treated with either vehicle (0.1% DMSO) or JV-1–36 (0.01–15 μM) (8 h). Our observations suggest that moderate concentrations of GHRHAnt (0.01–1 μM) do not affect cell viability. However, in higher concentrations, JV-1–36 (2–15 μM) reduced cell viability (Figure 1C).

### 3.4. Effects of H_2_O_2_ on A549 Cell Viability

A549 cells were treated with either vehicle (PBS) or H_2_O_2_ (0.001–2 mM) for 8 h. As shown in Figure 1D, H_2_O_2_ at 0.001–0.5 mM did not significantly affect A549. Those groups exposed to higher concentrations (1–2 mM) of that ROS generator depicted low viability levels.

### 3.5. Effects of H_2_O_2_ on HeLa Cell Viability

HeLa cells were exposed to vehicle (PBS) or H_2_O_2_ (0.001–2 mM) (8 h). At the concentrations of 0.001–0.1 mM, H_2_O_2_ did not affect cell viability, in contrast to the higher doses (0.5–2 mM) (Figure 1E).

### 3.6. Effects of GHRHAnt on ROS Generation in A549 and HeLa Cells

**Figure 2.**
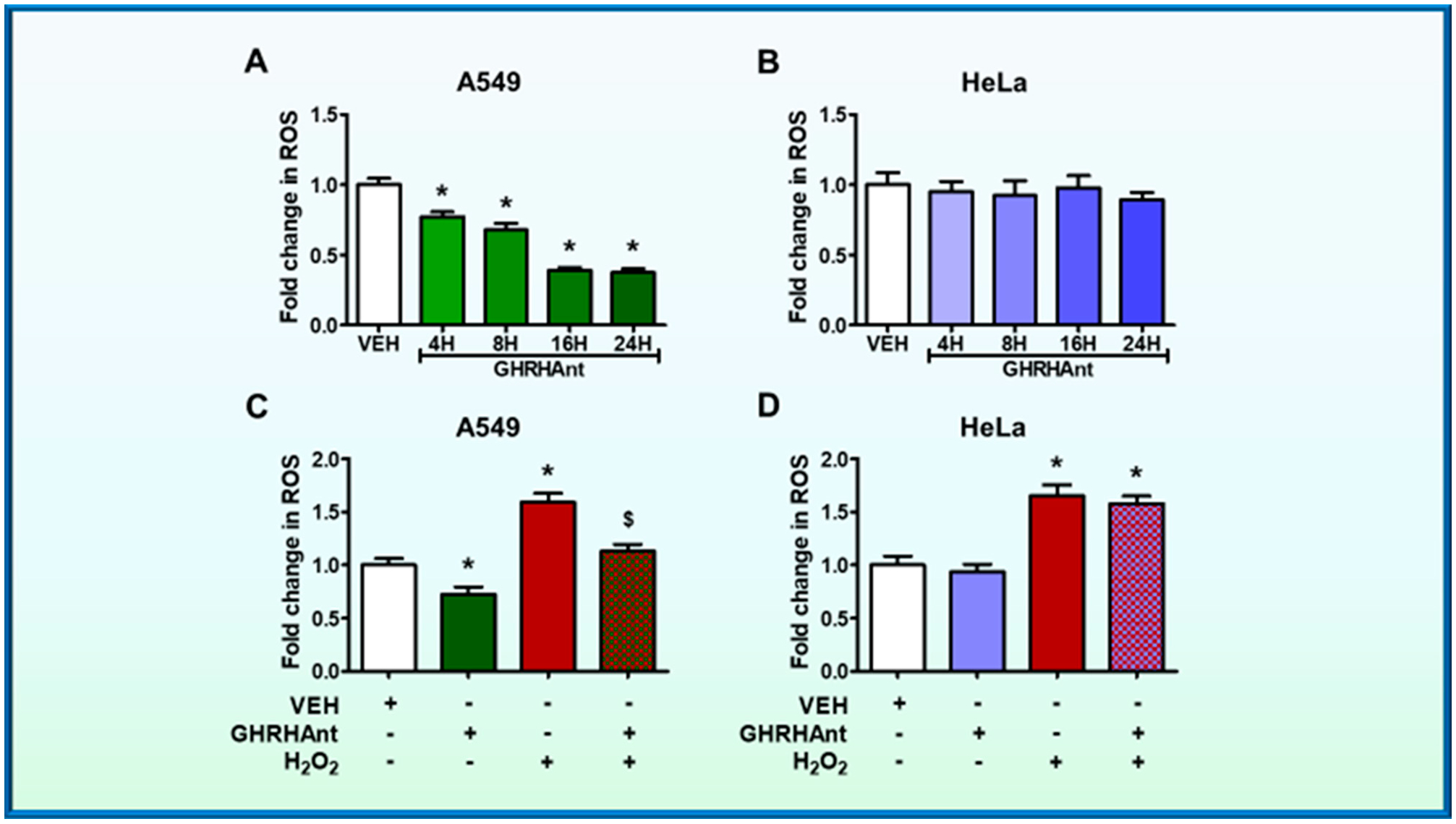
GHRHAnt protect against hydrogen peroxide (H_2_O_2_)-induced ROS generation. Microplate reader analysis of ROS in (**A**) A549 and (**B**) HeLa cells. The cells were treated with vehicle (VEH) (0.1% DMSO) or GHRHAnt (1 μM) for 4, 8, 16, and 24 h. The fold change in the ROS generation of GHRHAnt-treated cells was compared to the vehicle-treated cells. n = 4 per group. Means ± SEM. (**C**) A549 and (**D**) HeLa cells were pretreated with VEH (0.1% DMSO) or GHRHAnt (1 μM) for 8 h before VEH (PBS) or H_2_O_2_ (0.1 mM) exposure (8 h). The fold change in the ROS generation of the cells treated with H_2_O_2_ and/or GHRHAnt was compared to that of the cells treated with vehicle. n = 4 per group. Means ± SEM. * *p* < 0.05, vs. VEH; ^$^ *p* < 0.05 vs. H_2_O_2_.

Cells were exposed to either vehicle (0.1% DMSO) or GHRHAnt (1 μM) for 4, 8, 16, and 24 h. Our results demonstrate that GHRHAnt JV-1–36 inhibits ROS production in A549 cells (Figure 2A), while HeLa cells were unaffected (Figure 2B). The latter cell line does not express GHRH-R.

### 3.7. Effects of GHRHAnt on H_2_O_2_-Induced ROS Generation in A549 and HeLa Cells

To evaluate the effects of GHRHAnt JV-1–36 on H_2_O_2_-induced ROS generation, the cells were exposed to vehicle (0.1% DMSO) or JV-1–36 (1 μM) for 8 h prior to H_2_O_2_ (0.1 mM, 8 h) exposure. Our results demonstrate a significant induction of ROS by H_2_O_2_ in A549 cells, while GHRHAnt prevented that effect (Figure 2C). That antagonist did not affect the HeLa cells (Figure 2D).

## 4. Discussion

Reactive oxygen species (ROS) are partially reduced metabolites of oxygen, characterized by strong oxidizing capabilities. The majority of those radicals are produced by mitochondria as a result of oxidative phosphorylation. The electron transport chain is a mitochondrial pathway that encompasses five multimeric complexes. Complex I and III generate superoxide toward the matrix and intermembrane space [16]. ROS contribute to tumor development by enhancing cell survival, proliferation, protein synthesis, and glucose metabolism [12,17]. Moreover, these oxygen radicals are associated with inflammation progression. Superoxide anion (O^2−^) and hydrogen peroxide (H_2_O_2_) are second messengers capable of releasing growth factors, chemokines, and cytokines [18].

H_2_O_2_ is derived from membrane and mitochondrial sources, and it reversibly oxidizes cysteine residues (e.g., protein tyrosine phosphatases, protein tyrosine kinases, receptor tyrosine kinases) [19]. This ROS inducer is produced by epithelial cells during wound healing, and triggers the conversion of fibroblasts into myofibroblasts [20]. However, H_2_O_2_ production can impair DNA and cell metabolism, partially due to the Fenton reaction-induced hydroxyl radical (OH) [21]. At low concentrations, it mediates cell growth via mitogenic oxidase Nox1, a homologue of gp91phox [22]. In higher concentrations, H_2_O_2_ triggers pro-inflammatory mediators, including tumor necrosis factor (TNF-α) [23].

Cancer and inflammation are interrelated [24,25], whereas the latter condition exacerbates cardiovascular complications, diabetes, arthritis, Alzheimer’s disease, pulmonary, and autoimmune disorders [26]. GHRHAnt counteract metastasis [1,27,28], and A549 cells were utilized to assess the effects of those compounds in H_2_O_2_-induced ROS generation. HeLa cells were used to assess specificity, since they do not respond to GHRH (Figure 1A) [4].

GHRHAnt JV-1–36 significantly reduced ROS production in lung cancer cells (Figure 2A), whereas ROS generation in HeLa cells was unaffected (Figure 2B). HeLa cells do not respond to GHRH, since they lack the corresponding receptors (Figure 1A) [15]. Figure 2C demonstrates the significant induction of ROS by H_2_O_2_ in A549 cells and demonstrates that JV-1–36 exerted anti-oxidative effects in those cells. However, no changes were observed in HeLa cells (Figure 1D). Those outcomes align with previous observations on the anti-oxidative effects of another GHRHAnt, namely JMR132, in prostate cancers. It was reported that cyclooxygenase 2 (COX-2) and cytochrome c oxidase IV (COX-IV) were involved in those effects, since GHRHAnt suppressed their expression [17]. The anti-oxidative effects of GHRHAnt are not limited to malignancies. Indeed, the GHRHAnt MZ-5–156 was shown to reduce oxidative stress in aging mice brain, as reflected in the glutathione (GSH) and glutathione peroxidase (GPx) expression levels [29].

GHRHAnt can suppress inflammation in normal lung microvascular endothelial cells via redox modulation [30,31]. Highly reactive oxygen metabolites are associated with acute lung injury (ALI), acute respiratory distress syndrome (ARDS), asthma, and cystic fibrosis [31,32]. Superoxide dismutase produces H_2_O_2_ from superoxide anion, which produces hydroxyl radicals. Chronic exposure in H_2_O_2_ increases pulmonary artery pressure and vascular permeability, inflicting lung damage [32]. It has been previously reported that GHRHAnt suppress NF-κB [33], inducible nitric oxide synthase [34], and induces the tumor suppressor P53 [35]. This transcription factor reduces ROS production by controlling the expression of antioxidant genes such as superoxide dismutase 2 (SOD2), glutathione peroxidase 1 (GPX1), and catalase (CAT) [36].

P53 coordinates a complex framework to maintain cell homeostasis and genome stability [37]. In addition, it regulates redox homeostasis through transcriptional and non-transcriptional activities. P53 induction suppresses oxidative stress in the lung endothelium, as indicated by the reduced expression of the lipid peroxidation marker malondialdehyde (MDA) [24,30,31]. Moreover, NIMA-related kinases, or never-in-mitosis-A-related kinases (NEKs), are involved in the regulation of P53. NEK2, NEK3, NEK4, NEK7, and NEK9 upregulation has been observed in the lungs of septic mice [38]. NEK2 phosphorylates P53 at Ser315 and decreases its stability [39]. Suppression of this transcription factor by a high glucose (HG) levels is associated with HG-induced oxidative stress in endothelial cells, leading to endothelial damage and tissue injury [40]. GHRHAnt can induce P53 expression levels in both lung endothelial cells [15,41] and cancers [17], indicating that the antioxidant effects of those peptides may be mediated by P53, a UPR downstream target. However, additional studies are needed to interrogate the molecular cascades involved in those events.

UPR consists of protein kinase RNA-like ER kinase (PERK), inositol requiring enzyme 1α (IRE1α), and activating transcription factor 6 (ATF6) [42,43]. ATF6 was reported to participate in adaptive responses, so to ameliorate severe inflammatory disorders [44,45]. Perturbation of protein folding promotes the dissociation of binding immunoglobulin protein (BiP) or 78 kDa glucose-regulated protein (GRP-78) from the luminal domain of those sensors, which in turn activates UPR [44]. UPR can also mediate the protective effects of GHRHAnt in barrier dysregulation, a hallmark of the potentially lethal ARDS. GHRHAnt counteracted the kifunensine (UPR suppressor)-induced lung endothelial barrier dysfunction [31]. ARDS is responsible for the death of millions worldwide due to COVID-19; and targeted medicine for that disorder does not exist, so far. UPR can also positively regulate P53, and its modulation regulates barrier function [46]. Hence, GHRHAnt/UPR/P53 may form an autoregulatory loop to protect against barrier disorders. Further research will shed light on the role of UPR in the anti-oxidative activities of GHRHAnt in the P53 context. To do so, we will utilize genetically modified mice which express more or less of P53, and elevated UPR levels, as performed before [44,47,48].

Our observations reveal that JV-1–36 exerts anti-oxidative effects in A549 lung cancer cells. Considering the anti-inflammatory effects of GHRHAnt in normal lung endothelial cells, and their anti-oxidative activities in the inflamed endothelium [31], we conclude that GHRHAnt represent an exciting therapeutic possibility for the treatment of lung inflammatory diseases. Future studies will address the exact interrelations of GHRHAnt, UPR, and P53 in cancers, utilizing transgenic mice and genetically modified cancer cells.

## Data Availability

The data presented in this study are available on request from the corresponding author.
